# Genome-Wide Identification and Bioinformatics Characterization of Superoxide Dismutases in the Desiccation-Tolerant Cyanobacterium *Chroococcidiopsis* sp. CCMEE 029

**DOI:** 10.3389/fmicb.2021.660050

**Published:** 2021-05-28

**Authors:** Alessandro Napoli, Federico Iacovelli, Claudia Fagliarone, Gianmarco Pascarella, Mattia Falconi, Daniela Billi

**Affiliations:** Department of Biology, University of Rome Tor Vergata, Rome, Italy

**Keywords:** anhydrobiosis, astrobiology, superoxide dismutases, bioinfomratics, cyanobacteria

## Abstract

A genome-wide investigation of the anhydrobiotic cyanobacterium *Chroococcidiopsis* sp. CCMEE 029 identified three genes coding superoxide dismutases (SODs) annotated as MnSODs (SodA2.1 and SodA2.2) and Cu/ZnSOD (SodC) as suggested by the presence of metal-binding motifs and conserved sequences. Structural bioinformatics analysis of the retrieved sequences yielded modeled MnSODs and Cu/ZnSOD structures that were fully compatible with their functional role. A *signal*-*peptide bioinformatics* prediction identified a Tat signal peptide at the N-terminus of the SodA2.1 that highlighted its transport across the thylakoid/cytoplasmic membranes and release in the periplasm/thylakoid lumen. Homologs of the Tat transport system were identified in *Chroococcidiopsis* sp. CCMEE 029, and the molecular docking simulation confirmed the interaction between the signal peptide of the SodA2.1 and the modeled TatC receptor, thus supporting the SodA2.1 translocation across the thylakoid/cytoplasmic membranes. No signal peptide was predicted for the MnSOD (SodA2.2) and Cu/ZnSOD, thus suggesting their occurrence as cytoplasmic proteins. No FeSOD homologs were identified in *Chroococcidiopsis* sp. CCMEE 029, a feature that might contribute to its desiccation tolerance since iron produces hydroxyl radical *via* the Fenton reaction. The overall-overexpression in response to desiccation of the three identified SOD-coding genes highlighted the role of SODs in the antioxidant enzymatic defense of this anhydrobiotic cyanobacterium. The periplasmic MnSOD protected the cell envelope against oxidative damage, the MnSOD localized in the thylakoid lumen scavengered superoxide anion radical produced during the photosynthesis, while the cytoplasmic MnSOD and Cu/ZnSOD reinforced the defense against reactive oxygen species generated at the onset of desiccation. Results contribute to decipher the desiccation-tolerance mechanisms of this cyanobacterium and allow the investigation of its oxidative stress response during future space experiments in low Earth orbit and beyond.

## Introduction

Water removal induces damage at every level of the cellular organization, including membrane phase transition and oxidative damage to lipids, proteins, and DNA that are lethal to the majority of organisms (França et al., 2007). Notably a few species of bacteria, cyanobacteria, yeasts, plants, and small invertebrates are capable of surviving desiccation through a unique strategy known as anhydrobiosis (Crowe and Crowe, 1992). Upon water removal, anhydrobiotes enter a metabolic dormancy and return back to life thanks to mechanisms that avoid/limit desiccation-induced damages, among which antioxidant defense plays a crucial role (Rebecchi, 2013).

Among anhydrobiotic cyanobacteria *Chroococcidiopsis* strains isolated from hot and cold deserts have been extensively used to investigate the limit of microbial survival under laboratory simulated and space conditions (Billi, 2019). For example, *Chroococcidiopsis* strains survived prolonged and extreme desiccation, like 4 years of air-drying under laboratory conditions (Billi, 2009; Fagliarone et al., 2020) and 18-month exposure to space vacuum in low Earth orbit outside the international space station (Mosca et al., 2021). In response to air-drying *Chroococcidiopsis* sp. CCMEE 029 was reported to accumulate trehalose and sucrose, two non-reducing sugars used by anhydrobiotes to replace water molecules and thus preventing membrane phase transition and stabilizing dried cellular components (Fagliarone et al., 2020). Evidences for the presence of an efficient antioxidant defense in desert *Chroococcidiopsis* strains, including CCMEE 029, were provided by the absence of protein oxidation after exposure to desiccation and hydrogen peroxide treatment (Fagliarone et al., 2017), as well as by the reduced reactive oxygen species accumulation in cells air-dried for 4 years (Billi, 2009). However, despite the relevance of the antioxidant defense, its role in the desiccation tolerance of desert strains of *Chroococcidiopsis* remains to be elucidated.

A first line of defense against reactive oxygen species is provided by superoxide dismutases (SODs) that catalyze the disproportion of superoxide to peroxide and molecular oxygen (Miller, 2012). Comparative analyses of cyanobacterial genomes showed that they encode four types of SODs and that the various metalloforms are distributed differently: (i) copper/zinc (CuZnSOD) occurs rarely among cyanobacteria; (ii) nickel (NiSOD) is present in unicellular strains; (iii) filamentous cyanobacteria possess a combination of iron (FeSOD) and NiSOD or FeSOD and manganese (MnSOD); and (iv) filamentous, heterocyst-developing cyanobacteria have FeSODs and MnSODs (Priya et al., 2007).

The sub-cellular localization of SODs defines the protection of the various cellular components against oxidative stress. Notably, cyanobacteria are unique among prokaryotes for possessing membrane-bound MnSOD anchored to cytoplasmic and thylakoid membranes; Fe and NiSOD occur in the cytosol and Cu/ZnSOD is periplasmic (Atzenhofer et al., 2002; Li et al., 2002; Regelsberger et al., 2004). The MnSOD localization is determined by an N-terminal, hydrophobic, transmembrane helix tail and indeed, for the two MnSODs of *Plectonema boryanum*, N-terminal residues were hypothesized to act as a signal sequence, guiding the protein through or anchoring it to the thylakoid membranes (Campbell and Laudenbach, 1995). While in *Anabaena* sp. PCC 7120 the processing of the N-terminal of a membrane-targeted MnSOD was reported to release a soluble enzyme in the cytosol and in the periplasmic/thylakoid lumen (Raghavan et al., 2013).

Indeed, in cyanobacteria the presence of a thylakoid membrane system, in addition to the outer and cytoplasmic membranes, demands a complex system for protein transport and sorting (Frain et al., 2016). Evidence suggested that at the N-terminus membrane-targeted proteins have a signal sequence specific for two highly conserved routes: the Secretory (Sec) pathway and the Twin-Arginine translocation (Tat) pathway (Frain et al., 2016). The Sec pathway translocates proteins across the membrane in the unfolded state, whereas the Tat system transports folded proteins (Frain et al., 2016). In both the Sec and Tat machinery proteins are targeted for export by a signal sequence characterized by a basic n-region, a longer h-region of hydrophobic amino acids and a c-region with positively charged amino acids, the so-called Sec-avoidance motif, and the peptidase-recognition sequence for signal sequence removal and release of the mature protein (Frain et al., 2016). The genome mining of 85 cyanobacteria revealed that the majority encode a single gene set of the Sec system, whereas homologs of the Tat systems were identified in all the surveyed genomes (Russo and Zedler, 2020). After the transport *via* the Sec or Tat system, protein sorting to a different localization occurs through the signal recognition particle (SRP) pathway or *via* spontaneous insertion (Frain et al., 2016).

In this study, we performed a genome-wide identification of SOD-coding genes in the anhydrobiotic cyanobacterium *Chroococcidiopsis* sp. CCMEE 029, followed by sequence and structural bioinformatics analysis in order to identify metal-binding motifs, to predict signal peptides for sub-cellular localization and to reconstruct three-dimensional models. Since a Tat peptide signal was identified at the N-terminus of one ChSOD, the *Chroococcidiopsis* sp. CCMEE 029’s genome was mined for genes encoding proteins of the Tat transport system. A molecular docking simulation was performed to investigate the interaction between the Tat signal peptide of the ChSOD and the modeled TatC receptor. Finally, in order to unravel the role of the ChSODs in the desiccation response of *Chroococcidiopsis* sp. CCMEE 029, the expression of the identified SOD-coding genes was monitored by means of real-time quantitative PCR (RT-qPCR) during 10 and 60 min of desiccation.

## Materials and Methods

### Organism, Culture Conditions, and Desiccation

The cyanobacterium *Chroococcidiopsis* sp. CCMEE 029 isolated from lithic growth in the Negev Desert (Israel) is maintained at the Department of Biology, as part of the Culture Collection of Microorganisms from Extreme Environments (CCMEE) established by E. Imre Friedmann and Roseli Ocampo-Friedmann. The strain was grown under routine conditions at 25°C in liquid BG-11 medium under a photon flux density of 40 μmol/m^2^/s provided by fluorescent cool-white bulbs. Desiccation was performed by immobilizing approximately 10^8^ cfu of *Chroococcidiopsis* on 0.2 μm pore size hydrophilic polycarbonate membrane filters (Merck KGaG, Darmstadt, Germany) and stored for 10 and 60 min in a vacuum desiccator over silica gel (Sigma Aldrich, Saint Louis, MO, United States), at RT, in the dark. After treatment, samples were immersed in liquid nitrogen and stored at −80°C for further analyses.

### Identification of SOD-Coding Genes and Conserved Motifs

The genome sequence of *Chroococcidiopsis* sp. CCMEE 029 was obtained by using Illumina Solexa technology (CD Genomics, NY, United States) and SODs were identified using a Basic Local Alignment Search Tool (BLAST) using a protein query to scan a cyanobacterial protein database. The identification of conserved SOD motifs was performed using PROSITE signatures (Sigrist et al., 2012) and Pfam database (Finn et al., 2016). The prediction of Sec and Tat signal peptides was performed by using SignalP 5.0 server.^1^ The prediction of transmembrane helices in proteins was performed using TMHMM Server v. 2.0.^2^

### Multiple Sequence Alignment

Cyanobacterial SOD sequences were downloaded from NCBI and Uniprot databases (Leinonen et al., 2004) and multiple alignments were obtained by using Clustal Omega (Sievers and Higgins, 2014) based on HMM profile-profile techniques to generate sequence alignments. Sequence alignments were displayed graphically using JalView 2.10.5 (Waterhouse et al., 2009).

### Molecular Modeling of Proteins

In the absence of experimentally determined structures, two computational approaches were used to model the 3D structures of SODs and TatC protein. In detail, the sodA2.2 was modeled using the Swiss-Model web server (Waterhouse et al., 2018), using as a template the MnSOD structure (PDB ID: 1GV3) from *Nostoc* sp. PCC 7120 (Atzenhofer et al., 2002) whose sequence shared about 66% of identity with the query. The sodC was modeled using as a template the Cu/ZnSOD structure (PDB ID: 3KBF) from *Caenorhabditis elegans*, sharing about 40% of identity with the query (to be published). Since a suitable structural template for homology modeling the full-length SodA2.1 protein was not found, the protein sequence was submitted to the I-TASSER server, which exploits both threading and *ab initio* methods to build the models, reassembling structural fragments from threading templates using replica exchange Monte Carlo simulations (Yang and Zhang, 2015). Finally, TatC was modeled using as a template the twin arginine translocase receptor structure (PDB ID: 4HTS) from *Aquifex aeolicus* (Ramasamy et al., 2013), which shares about 37% of identity with our query sequence.

### Refinement of I-TASSER-Generated Models Through Molecular Dynamics Simulations

The SodA2.1 topology and coordinate files were generated using the Amber 16.0 (Case et al., 2005) tLeap tool, parameterizing the proteins with the Amber ff19SB force field (Tian et al., 2020). The system was solvated with TIP3P water (Jorgensen et al., 1983) in a cubic box with 14 Å between the protein surface and the box boundaries, and electrostatically neutralized by the addition of appropriate number of counterions. The GPU-enabled PMEMD module of Amber 16.0 package was used to perform the molecular dynamics simulations. The system was previously energy minimized for 2,000 steps of steepest descent algorithm, followed by 500 steps of conjugate gradient algorithm to eliminate close van der Waals contacts generated by the modeling procedure and then gradually heated from 0 to 300 K in 1 ns, followed by constant pressure equilibration at 300 K for 1 ns. Following this phase, 50 ns production MD runs were carried out with periodic boundary conditions in the NPT ensemble, at a temperature of 300°K using Langevin thermostat (Feller et al., 1995) and a constant pressure of 1.0 atm with isotropic molecule-based scaling. Bond lengths involving bonds to hydrogens have been constrained using the SHAKE algorithm (Ryckaert et al., 1977). Long-range electrostatic forces were calculated using the particle-mesh Ewald (PME) method (Toukmaji et al., 2000). The 50-ns trajectory was subjected to clustering procedures, to extract representative structures for the sodA2.1 protein, using the GROMACS 2019.3 (Abraham et al., 2015) gmx cluster tool, selecting the gromos method (Daura et al., 1999) and imposing a 0.12 nm cut-off for the geometrical clustering.

### Molecular Docking

The molecular docking between TatC and sodA2.1 was modeled through the HADDOCK 2.4 web server using the advanced interface (van Zundert et al., 2016), with the following parameters: number of structures for rigid bodies was set to 5,000, number of structures for semi-flexible refining was set to 500 and number of structures for the explicit solvent refinement to 250. To guide the docking between the two proteins, distance restraints were specified between the E46-Oε2.TatC and R6-NH2.SodA2.1 and between the E129-Oε1.TatC and R5-NH1.SodA2.1 atoms. A second docking procedure was also performed imposing the distance restraints between the E129-Oε1.TatC and R6-NH2.SodA2.1 and between the E46-Oε2.TatC and R5-NH1.SodA2.1 atoms, to check for the most reliable pose. The resulting complexes were analyzed through the UCSF Chimera 1.13 visualization program (Pettersen et al., 2004).

### RNA Extraction and Gene Expression Analysis

Total RNA was extracted from samples exposed to 10 and 60 min of desiccation and from liquid, control cultures by using 1 ml of TRI Reagent (Sigma Aldrich, Saint Louis, MO, United States) followed by treatment with RQ1 RNase-Free DNase I (Promega Corporation, Madison, WI, United States) according to the manufacturer’s instructions. Then, 1 μg of total RNA for each sample was retrotranscribed to first-strand cDNA by using the SensiFASTTM cDNA Synthesis Kit (Bioline Meridian Life Science Memphis, TN, United States). Real-time reactions were performed in 12 μl, including 1 μg of cDNA template, 6 μl of iTaqTM universal SYBR® Green supermix (BioRad Laboratories, Hercules, CA, United States) and 400 nM of the appropriate primer (Table 1). Primer specificity was confirmed by melting curve analysis. 16S rRNA (GenBank accession number AF279107) was used as a reference gene. PCR cycling conditions were performed in a StepOnePlusTM Real-Time PCR System (Thermo Fisher Scientific, Waltham, MA, United States) as follows: a cycle of 95°C for 10 min, then 40 cycles of 95°C for 15 s, and 60°C for 1 min, followed by a ramp from 60 to 95°C for melting curve stage. Relative mRNA levels were calculated by the comparative cycle threshold (Ct) method. Values obtained for liquid controls were set as 1 and values of dried cells were considered to be upregulated (>1) or downregulated (<1). For each gene target *n* ≥ 3 qPCR reactions were conducted, each one in duplicate.

**Table 1:** Primers used for real-time quantitative PCR (RT-qPCR).

Gene	PCR primers	Sequence (5'-3')	PCR product size (bp)
*16S rDNA*	chr16S-F chr16S-R	TACTACAATGCTACGGACAA CCTGCAATCTGAACTGAG	83
*sodA2.1*	chrsodA2.1-F chrsodA2.1-R	TAGTTGTAATCTGGAGTCT GCCTCTGCTATCAATAAG	136
*sodA2.2*	chrsodA2.2-F chrsodA2.2-R	TTGCCGAATTGATTACGA CTGCTACGAACTAACCAA	112
*sodC*	chrsodC-F chrsodC-R	ACACTCCTCTTACGAATGC CCTCCTGCCTCATACTCA	75

## Results

### Identification of SOD-Coding Genes

Three genes, namely *sodA2.1*, *sodA2.2*, and *sodC*, were identified in the genome of *Chroococcidiopsis* sp. CCMEE 029 that encoded three ChSODs annotated according to KEGG as Fe/MnSOD and Cu/ZnSOD (Table 2).

**Table 2:** SOD-coding genes in *Chroococcidiopsis* sp. CCMEE 029.

Gene	Length (nt)	KEGG	Species with highest BlastP similarity (%)	Genbank accession number
*sodA2.1*	789	K04564 SOD2, Fe-Mn family	*Fischerella* sp. PCC 9605 (72.80%)	MW422282
*sodA2.2*	612	K04564 SOD2, Fe-Mn family	*Chroococcidiopsis* sp. TS-821 (83.17%)	MW422283
*sodC*	684	K04565 SOD1, Cu-Zn family	*Scytonema hofmannii* FACHB-248 (58.24%)	MW422284

The *sodA2.1* gene was 789-bp long and the codified protein showed the highest similarity with the Mn/FeSOD from *Fischerella* sp. PCC 9605 (NCBI Reference Sequence: WP_026733564.1; BlastP output: query cover 99%, e-value 5e-140, and total score 403). The *sodA2.2* gene had a length of 612-bp coding a protein with the highest similarity with the Mn/FeSOD from *Chroococcidiopsis* sp. TS-821 (NCBI Reference Sequence: WP_104545601.1; BlastP output: query cover 99%, e-value 2e-129, and total score 372). The *sodC* gene had a length of 684-bp coding a SOD with the highest similarity with Cu/ZnSOD from *Scytonema hofmannii* FACHB-248 (NCBI Reference Sequence: MBD2608087.1; BlastP output; query cover 78%, e-value 2e-66, and total score 213).

### Multiple Sequence Alignment and SOD Conserved Motifs

**Figure 1:**
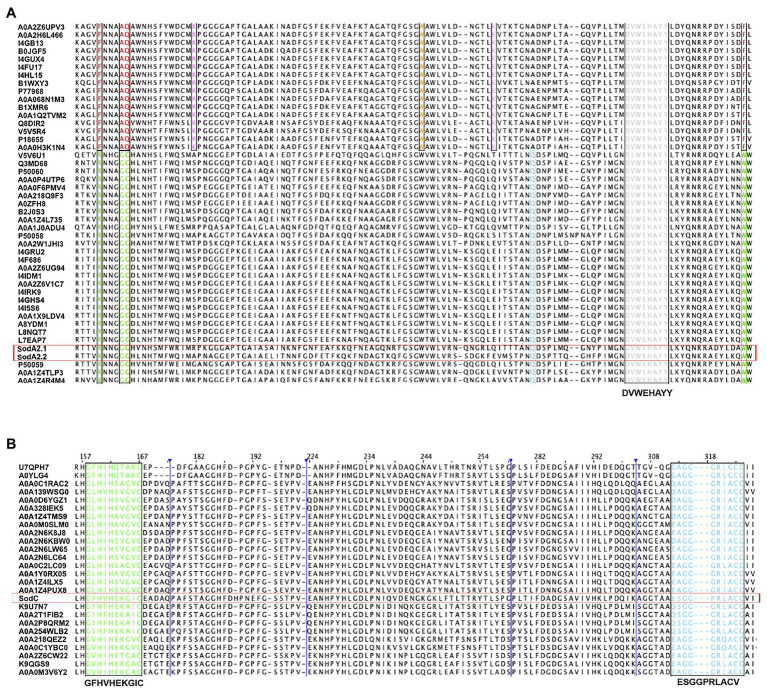
Multiple alignment of superoxide dismutases (SODs) from *Chroococcidiopsis* sp. CCMEE 029. **(A)** First quartile sequence of MnSODs (SodA2.1 and SodA2.2) and cyanobacterial Fe/MnSOD. Metal-binding motif DVWEHAYY is highlighted in gray. Highly conserved metal specific residues are highlighted in green for MnSODs and in red for FeDODs. Residues involved in outer sphere hydrogen bonding are highlighted in cyan for MnSODs and for FeSODs in orange. Lys (K) residue of FeSODs involved in photosynthetic context is boxed in pink. **(B)** Multiple alignments of half sequence of CuZnSod (SodC) and cyanobacterial CuZnSods. The copper domain G-F-H-[ILV]-H-x-[NGT]-[GPDA]-[SQK]-C is highlighted in green and the conserved motif G-[GA]-G-G-[AEG]-R-[FIL]-[AG]-C-G is in blue. The list of UniProt entries was provided in the Supplementary Tables S1, S2.

The multiple sequence alignment of the two ChSODs (SodA2.1 and SodA2.2) and a selection of previously identified cyanobacterial SODs (Priya et al., 2007) revealed the presence in the SodA2.1 and SodA2.2 of the cyanobacterial metal-binding motif DVWEHAYY (Pfam:PF00081;PF02777; Figure 1). In addition, the two ChMnSODs showed the following features used to differentiate MnSOD from FeSOD (Priya et al., 2007): (i) the metal specific tryptophan residue specific; (ii) the glutamine residue involved in outer sphere hydrogen bonding; and (iii) the absence of two lysine residues occurring in FeSODs (Figure 1A).

**Figure 2:**
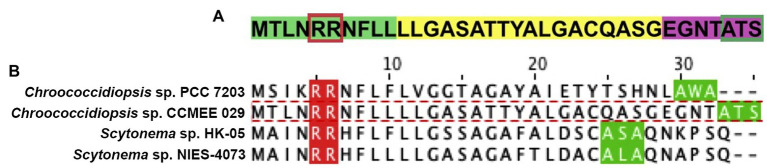
Predicted Tat signal peptide at the N-terminal of MnSOD (SodA2.1) of *Chroococcidiopsis* sp. CCMEE 029. **(A)** Tripartite structure with the positively charged n-region (highlighted in green) and twin-arginine dipeptide (boxed in red), the central hydrophobic h-region (yellow) and the c-region with peptidase recognition site (purple) and cleavage site (boxed in green). Alignment of SodA2.1 of *Chroococcidiopsis* sp. 029 and cyanobacterial MnSOD with a Tat signal peptide. **(B)** *Chroococcidiopsis* sp. PCC 7203 (UniProt entry K9TVN2), *Scytonema* sp. HK-05 (UniProt entry A0A1Z4IPS3) and *Scytonema* sp. NIES-4073 (UniProt entry A0A1Z4PNX6); twin-arginine dipeptide (red) and signal sequence cleavage site (green).

The multiple sequence alignment of the Cu/ZnSOD (SodC) and a selection of cyanobacterial Cu/ZnSOD showed the presence of GFHVHEKGIC and ESGGPRLACV sequences corresponding to the copper domain (Pfam:PF00080) G-F-H-[ILV]-H-x-[NGT]-[GPDA]-[SQK]-C, and the conserved motif G-[GA]-G-G-[AEG]-R-[FIL]-[AG]-C-G, respectively (Figure 2B).

### Characterization of the N-Terminus of ChSODs

The bioinformatics analysis of the N-terminus of the SodA2 of *Chroococcidiopsis* sp. CCMEE 029 using the Prosite and SignalP 5.0 tools predicted the absence of signal peptides, thus suggesting that this cytoplasmic localization. No signal peptides were predicted for the N-terminus of the SodC, suggesting that also the CuZnSOD was soluble in the cytoplasm.

On the contrary, the Prosite tool predicted a Tat signal peptide (Prosite entry PS51318) at the N-terminal of the SodA2.1, with a tripartite structure consisting of a positively charged segment (n-region) with the twin-arginine dipeptide, a central hydrophobic domain (h-region) and a c-terminal (c-region) with the presence of positively charged amino acids, the so-called Sec-avoidance motif, and the signal peptidase cleavage site (Figure 2A). The SignalP 5.0 tool confirmed the presence of a Tat signal peptide in the SodA2.1 with a likelihood-score value of 0.6165, thus suggesting its translocation across the periplasmic/thylakoid membranes.

The multiple sequence alignment of the N-terminus of the SodA2.1 of *Chroococcidiopsis* sp. CCMEE 029 and a selection of cyanobacterial MnSOD possessing a predicted Tat signal peptide revealed substitutions in the consensus sequence S/Thr-R-R-x-F-L and in the Ax-A cleavage site. In particular, the SodA2.1 exhibited the substitution of the Ser residue (S) with an Arg (N) residue in the consensus sequence, and the substitution of the Ala (A) residue with a Ser (S) residue in the signal peptidase cleavage site (Figure 2B).

Furthermore, the TMHMM method predicted the absence of transmembrane helices in SodA2.1, SodA2.2, and SodC of *Chroococcidiopsis* sp. CCMEE 029.

### Identification of *tat* Genes

The *in silico* analysis of *Chroococcidiopsis* sp. CCMEE 029’s genome identified three genes encoding Tat-system proteins identified by KEGG as two TatA proteins and one TatC protein (Table 3). The *tatA(1)* gene was 273-bp long coding a protein with the highest similarity with the ortholog of *Nostoc* sp. NMS1 (NCBI Reference Sequence MBN3910739.1; query cover 100%, e-value 5e-45, and total score 150). The *tatA(2)* gene was 180-bp length coding a protein with the highest similarity with the ortholog of *Scytonema* sp. NIES-4073 (NCBI Reference Sequence: WP_096568605.1; BlastP output: query cover 88%, e-value 6e-24, and total score 94.4). The *tatC* gene was 798-bp long coding a protein with the highest similarity with the ortholog of *Chroococcidiopsis* sp. FACHB-1243 (NCBI Reference Sequence MBD2306943.1; BlastP output; query cover 100%, e-value 2e-152, and total score 436). The survey of *Chroococcidiopsis* sp. CCMEE 029’s genome did not identify any orthologs of the *tatE* gene.

**Table 3:** Tat system genes of *Chroococcidiopsis* sp. CCMEE 029.

Gene	Length (nt)	KEGG	Species with highest BlastP similarity (%)	Genbank accession number
*tatA(1)*	273	K03116 Sec-independent protein translocase protein TatA	*Nostoc* sp. NMS1(81.11%)	MW422286
*tatA(2)*	180	K03116 sec-independent protein translocase protein TatA	*Scytonema* sp. NIES-4073 (88.46%)	MW422287
*tatC*	798	K03118 Sec-independent protein translocase protein TatC	*Chroococcidiopsis* sp. FACHB-1243 (81.58%)	MW422285

### Multiple Sequence Alignment and Conserved Sequence Motifs of ChTatA Proteins

**Figure 3:**
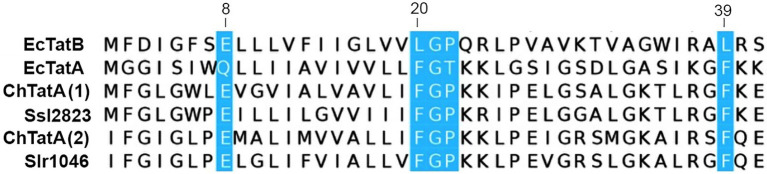
Alignment of TatA proteins from *Chroococcidiopsis* sp. CCMEE 029. ChTat1 and ChTat2 aligned with homologs from *Synechocystis* sp. PCC 6803 (Slr1046 and Ssl2823) and *Escherichia coli* (EcTatA and EcTatB). Conserved residues of EcTatA, EcTatB are highlighted in blue. Numbering corresponds to EcTatA and EcTatB.

The alignment of the two TatA proteins of *Chroococcidiopsis* sp. CCMEE 029 (ChTat1 and ChTat2) with homologs from *Synechocystis* sp. PCC 6803 and *Escherichia coli* (Figure 3) revealed that like the homolog from *Synechocystis* sp. PCC 6803, the two ChTatA proteins have residues that discriminate between EcTatA and EcTatB of *E. coli* (Hicks et al., 2003). Like EcTatA, the two ChTatA have a Phe residue (F) immediately before the hinge Gly residue (G), corresponding to position 20 of EcTatA and a Phe residue (F) corresponding to position 39 of EcTatA. While like EcTatB, the two ChTatA proteins have a Pro residue (P) immediately after the hinge Gly residue (G) and a Glu residue (E) in the predicted transmembrane domain, corresponding to position 8 of EcTatB.

The bioinformatics analysis carried out using the predictor TMHMM revealed that the two ChTatA have a single putative transmembrane helical-spanning domain, while *Ch*TatC protein contains six putative transmembrane helical-spanners. For ChTatC, the Prosite tool predicted the presence of the YFEFVLLLLFSTGLAFQIPI sequence corresponding to the TatC family signature (Prosite: PS01218) Y-x(2)-[FL]-[LIVMAFNT]-[LIVMAFT]-x-[LVSI]-x(4)-[GASF]-x(2)-F-[EQ]-[LIVMFC]-P-[LIVM].

### Molecular Models of ChSODs Structures

**Figure 4:**
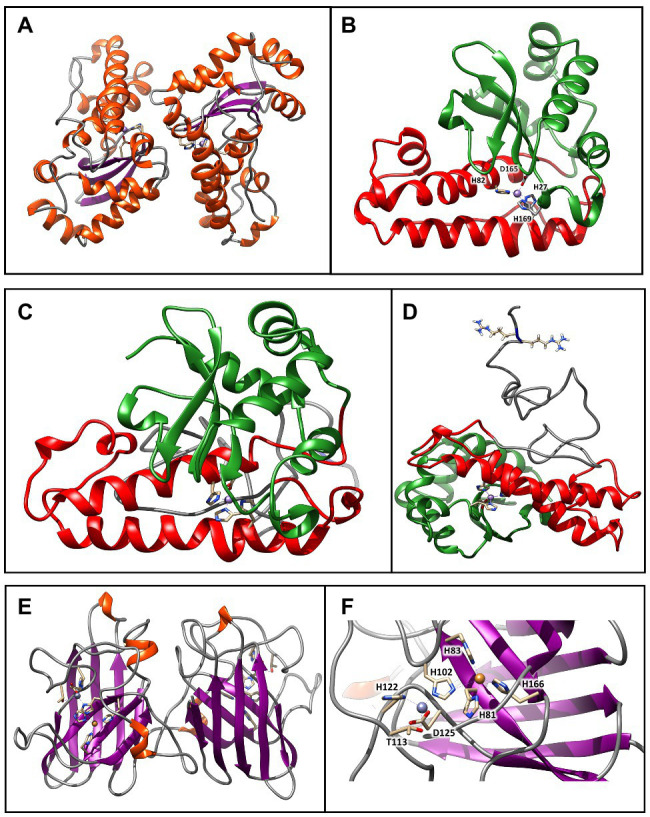
Molecular models of SODs from *Chroococcidiopsis* sp. CCMEE 029. **(A)** Model of the SodA2.2 dimer generated using as a template the crystal structure of MnSOD from *Nostoc* sp. PCC 7120 (PDB ID: 1GV3). The β-strands are represented by violet arrows, while the α-helices and the loops are shown as orange spirals and light gray wires, respectively. **(B)** The red and green colors indicate the N-terminal helical hairpin domain and the C-terminal α/β domain. The metal binding site is shown in stick representation. **(C)** Model of the sodA2.1 generated by the I-TASSER structure prediction algorithm. The red and green colors indicate the N-terminal helical hairpin domain and the C-terminal α/β domain typical of bacterial MnSOD. **(D)** The unstructured N-terminal region (gray wire) holds the two sequential arginines required for the interaction with the TatC receptor. The metal-binding site and the arginines are shown in stick representation. **(E)** Model of the SodC dimer generated using as a template the crystal structure of Cu/ZnSOD from *Caenorhabditis elegans* sp. (PDB ID: 3KBF). The β-strands are represented by violet arrows, while the α-helices and the loops are shown as orange spirals and light gray wires, respectively. **(F)** The copper (orange atom) and zinc (plum atom) binding sites are shown in stick representation. These images were generated using the Chimera program.

The three-dimensional structure obtained through homology modeling of the SodA2.2 sequence obtained using as a template the MnSOD from *Nostoc* sp. PCC 7120 (Atzenhofer et al., 2002), sharing about 66% of identity with the query (Figure 4A). The modeled SodA2.2 protein consisted in a homodimeric protein with each subunit being divided in two distinct domains: a N-terminal helical hairpin domain (Figure 4B, red) and a C-terminal α/β domain (Figure 4B, green), containing three-stranded antiparallel β-sheet and five α-helices. In the obtained model, the highly conserved MnSOD metal-binding site connected both domains *via* the metal ion, which binds the His27 and His82 of the N-terminal domain and Asp165 and His169 of the C-terminal domain.

The three-dimensional structure of the SodA2.1 was modeled using the I-TASSER pipeline (Yang and Zhang, 2015), since the N-terminal portion of the molecule could not be homology modeled due to the lack of experimental data. Starting from the five different models generated by I-TASSER, the one with the better C-score was selected, that was subjected to an optimization procedure through 50 ns long classical molecular dynamics simulation to solve modeling issues (Figure 4C). As shown, the structure obtained by geometrical clustering of the molecular dynamics trajectory is coherent with the typical fold of a bacterial MnSOD (Figure 4C), with the presence of an unstructured region at the N-terminus, which contains the predicted TAT signal peptide. The reference structure extracted from the trajectory, representative of about the 50% of the simulation time, indicates that the N-terminal region is arranged in the solvent so that the two arginines required for the interaction with TatC (Berks et al., 2014) remain easily accessible (Figure 4D).

The SodC was modeled using as a template the Cu/ZnSOD structure (PDB ID: 3KBF) from *C. elegans* (to be published), sharing about 40% of identity with the query. As shown in Figure 7 its three-dimensional structure was consistent with the classical eight-stranded *Greek key* β-barrel, with the active site held between the barrel and two surface loops (Figure 4E). The two subunits were tightly joined back-to-back, mostly by hydrophobic and some electrostatic interactions. The ligands of the copper and zinc were five histidine and one aspartate sidechains, with the zinc ion coordinated by His102, His122, and Asp125 and the copper by His81, His83, His102, and His166 (Figure 4F). The binding site was not fully conserved, since one of the conserved histidines usually coordinating the zinc ion was here replaced by a threonine sidechain.

**Figure 5:**
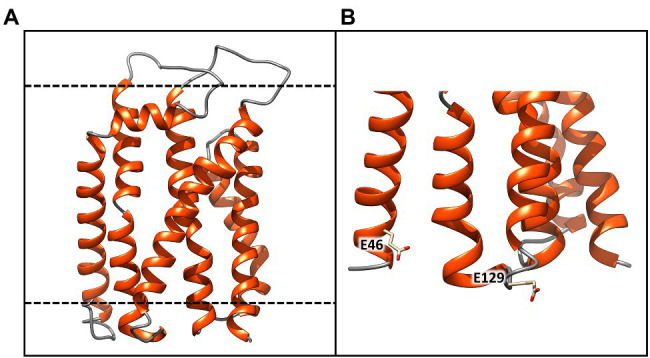
Molecular model of the TatC receptor of *Chroococcidiopsis* sp. CCMEE 029. **(A)** Model generated using as a template the crystal structure of twin arginine translocase receptor from *Aquifex aeolicus* (PDB ID: 4HTS). α-helices and loops are represented by orange spirals and light gray wires, respectively. **(B)** The dashed lines indicate the transmembrane region Glu46 and Glu129 would coordinate the positively charged guanidinium groups of the sequential arginine residues of the Tat signal peptide. This image was generated using the Chimera program.

**Figure 6:**
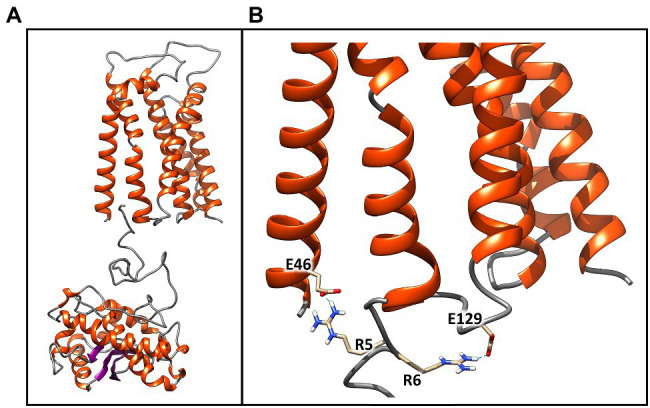
Best molecular complex between the TaTc receptor and the SodA2.1 protein of *Chroococcidiopsis* sp. CCMEE 029. The β-strands are represented by violet arrows, while the α-helices and the loops are shown as orange spirals and light gray wires, respectively. The structure accounts for the 98% of the total complexes generated by the HADDOCK webserver **(A)**. Detail of the interactions established between the Glu46–Glu129 and the positively charged guanidinium groups of the sequential arginine residues Arg5 and Arg6 of Tat signal peptide. Glutamic acid and arginine are shown in stick representation **(B)**. This image was generated using the Chimera program.

**Figure 7:**
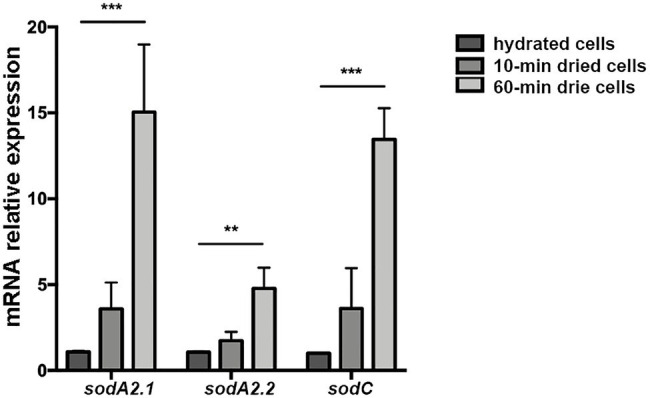
Expression of SOD-coding genes of *Chroococcidiopsis* CCMEE 029 in response to desiccation. The *sodA2.1* and *sodA2.2* genes encode two MnSODs and *sodC* gene encodes a CuZnSOD. Expression levels are shown as a ratio between the target gene and the 16S rRNA gene. Control: hydrated cells. Data represent mean ± SD (*n* ≥ 3), ^***^p < 0.001 and ^**^p < 0.01.

### Molecular Model of ChTatC Structure

The three-dimensional structure of the ChTatC protein was obtained from the homology modeling of the twin arginine translocase receptor from *A. aeolicus* (Figure 5). The modeled protein showed six membrane-spanning helices with the longest dimension of about 55 Å, essentially the same as the bilayer width, resulting in a small portion of the protein exposed outside the membrane (Figure 5A, black lines). However, the two negatively charged amino acids Glu46 and Glu129 would be appropriately positioned to coordinate the positively charged guanidinium groups of the sequential arginine residues of the Tat signal peptide (Figure 5B).

### Molecular Docking Between ChSodA2.1 and ChTatC Models

To further confirm the validity of the models, the structures of the SodA2.1 and TatC proteins were subjected to molecular docking simulations through the HADDOCK 2.4 web server to verify whether the interaction between the TAT signal peptide and its TatC receptor could occur as hypothesized in literature (Berks et al., 2014). The docking procedure was carried out imposing unambiguous distance restraints, forcing the interaction of Glu46 of TatC and Arg6 of sodA2.1 and of Glu129 of TatC with the Arg5 of sodA2.1, and vice versa. The obtained results confirmed that the two proteins can interact as previously described (Berks et al., 2014; Figure 6A). The two negatively charged amino acids Glu46 and Glu129 are located at the correct distance to interact through hydrogen bonds with the positively charged guanidinium groups of the sequential arginines of the signal peptide (Figure 6B).

### Expression Analysis of ChSOD-Coding Genes in Response to Desiccation

When cells of *Chroococcidiopsis* sp. 029 were exposed to 10 and 60 min of desiccation, the three SOD-encoding genes exhibited an overall over-expression. The *sodA2.1* gene encoding a MnSOD with the Tat signal peptide, showed mRNA levels higher than those of control liquid cultures at both the experimental points. Also the expression of the *sodC* gene encoding a cytoplasmic CuZnSOD was increased after 10- and 60-min desiccation when compared to control liquid cultures (Figure 7). While the *sodA2.2* gene encoding a cytoplasmic MnSOD showed a significant increase in the mRNA level only after 60 min of water removal compared to control liquid cultures, this value being lower than that of the *sodA2.1* and *sodC* genes at the same experimental point (Figure 7).

## Discussion

In order to unravel the role of SODs in the antioxidant defense of the anhydrobiotic cyanobacterium *Chroococcidiopsis* sp. CCMEE 029, a survey of its genome was carried out and three SOD-coding genes were identified. Two genes codified two ChMnSODs (SodA2.1 and SodA2.2) characterized by the metal binding motif DVWEHAYY that was reported to distinguish cyanobacterial SODs from other prokaryotic SODs (Priya et al., 2007). In addition, the ChMnSODs were unequivocally discriminated from FeSOD for the presence of the highly conserved metal residues as previously reported (Priya et al., 2007). The third identified gene codified a CuZnSOD (SodC) as revealed by the presence of the copper binding metal motif GFHVHEKGIC and the conserved motif ESGGPRLACV (Priya et al., 2007).

The structural bioinformatics analysis highlighted a compatibility between the retrieved ChSOD sequences and the modeled structures, thus confirming the presence of these detoxifying enzymes in *Chroococcidiopsis* sp. CCMEE 029. The SodA2.2 showed a typical fold as reported for *Anabaena* sp. PCC 71020 (Atzenhofer et al., 2002), consisting in a homodimeric protein with each having a N-terminal helical hairpin domain and a C-terminal α/β domain containing three-stranded antiparallel β-sheet and five α-helices. Due to the absence of experimentally determined structures, the three-dimensional structure of the SodA2.1 was modeled using the I-TASSER pipeline (Yang and Zhang, 2015) and the obtained structure was consistent with the typical fold of a bacterial MnSOD. The sodC was modeled using as a template the Cu/ZnSOD structure from *C. elegans* (to be published), revealing that the binding site was not fully conserved, since one of the conserved histidines in the zinc coordination was replaced by a threonine.

The BLAST analysis showed high similarities between ChMnSODs and homologs from filamentous cyanobacteria. This was in agreement with the similarities reported for DNA repair proteins of *Chroococcidiopsis* sp. CCMEE 029 (Mosca et al., 2019, 2021) and trehalose and sucrose biosynthetic enzymes (Fagliarone et al., 2020). Indeed, the unicellular *Chroococcidiopsis* genus and filamentous, heterocyst-differentiating cyanobacteria were reported to be each other’s closest living relatives (Fewer et al., 2002).

To get insights into the sub-cellular localization of the ChSODs a *signal*-*peptide bioinformatics* prediction was performed. The SignalP 5.0 tool allows the discrimination between three types of signal peptides: (i) Sec/SPI secretory signal peptides transported by the Sec translocon and cleaved by signal peptidase I; (ii) Sec/SPII lipoprotein signal peptides transported by the Sec translocon and cleaved by signal peptidase II; and (iii) Tat/SPI Tat signal peptides transported by the Tat translocon and cleaved by signal peptidase I (Almagro Armenteros et al., 2019). At the N-terminus of the SodA2.1 of *Chroococcidiopsis* sp. CCMEE 029A a Tat signal peptide was predicted showing the canonical tripartite structure with the typical twin-arginine peptide and the cleavage site for signal peptidase I (von Heijne, 1984). However, compared to the bacterial consensus Tat motif S/T-R-R-x-F-L-K (Berks, 1996) the Tat motif of SodA2.1 lacked the S/T residue and the Lyn (K) residue was replaced by a Leu (L) residue. Indeed, it was reported that the Ser/Thr (S/T) and Lyn (K) residues are not conserved in the Tat motifs of several cyanobacteria (Barnett et al., 2011). In addition, the SodA2.1 of *Chroococcidiopsis* sp. CCMEE 029 exhibited in the Ala-x-Ala peptidase cleavage site the substitution of an Ala (A) residue with a Ser (S) residue, a substitution considered acceptable for the peptidase binding (von Heijne, 1984).

The presence of the Tat signal peptide at the N-terminus of the SodA2.1 of *Chroococcidiopsis* sp. CCMEE 029 suggested its transport by the Tat machinery as a folded protein across the thylakoid/cytoplasmic membranes followed by its release in the periplasm/thylakoid lumen after the signal peptide cleavage. The release of the SodA2.1 in the periplasm/thylakoid lumen after its transport across the membranes was supported by the lack of predicted transmembrane-helical spanners. Moreover, the alignment of the N-terminus of the SodA2.1 and membrane-bound MnSOD from *Anabaena* sp. PCC 7120 (Regelsberger et al., 2002) did not reveal any shared conserved regions at the N-terminal (not shown). The structural bioinformatics analysis fully reconstructed the three-dimensional structure of the ChTatC showing six membrane-spanning helices as the majority of the TatC homologs (Rollauer et al., 2012). Moreover, the molecular docking simulation confirmed the interaction between the signal peptide of the SodA2.1 and the modeled TatC receptor, thus supporting the translocation of this ChMnSOD across the thylakoid/cytoplasmic membranes.

It was emphasized that the transport of folded proteins might be advantageous under environmental stressing conditions (Frain et al., 2016). Therefore, in *Chroococcidiopsis* sp. CCMEE 029 the transport of folded MnSOD in the periplasm might contribute to its desiccation tolerance by protecting the cell envelope against superoxide formed outside the cytoplasm. While the export of folded MnSOD in the thylakoid lumen might enable the sequestration of superoxide anion radical generated by an impaired oxygenic photosynthetic activity during the onset of water removal.

The *in silico* analysis of *Chroococcidiopsis* sp. CCMEE 029’s genome identified three genes encoding Tat machinery proteins: two TatA and one TatC. The presence of only one TatC was in agreement with the results of a survey of 85 cyanobacterial genomes revealing that half of them, including many *heterocyst*-forming cyanobacteria, have two TatA/B homologs, and the other half, including most of the marine picocyanobacteria, have one TatA/B homolog (Russo and Zedler, 2020). As reported for other cyanobacteria (Russo and Zedler, 2020), the two TatA from *Chroococcidiopsis* sp. CCMEE 029 were highly homologous to each other, and therefore, it was impossible to establish whether it encodes a TatABC or a minimal TatAC system (Russo and Zedler, 2020). Furthermore, the two ChTatA proteins exhibited residues that are exclusive to either TatA or TatB in *E. coli* (Hicks et al., 2003.) Therefore, as concluded for *Synechocystis* sp. PCC 6803 (Aldridge et al., 2008), it remained unclear whether *Chroococcidiopsis* sp. CCMEE 029 has a single TatABC system operating in both the thylakoid and cytoplasmic membranes or two minimal TatAC pathways operating independently in the two membrane locations.

No signal peptide was predicted at the N-terminus of the ChMnSOD codified by the *sodA2.2* gene, nor at the N-terminus of the ChCu/ZnSOD codified by the *sodC* gene, thus suggesting a cytoplasmic localization. The presence of a Cu/ZnSOD in *Chroococcidiopsis* sp. CCMEE 029 is remarkable because this enzyme is rare among cyanobacteria, as revealed by the survey of 64 genomes revealing its presence only in six *Synechococcus* strains and one *Lyngbya* strain (Priya et al., 2007).

The coexistence of MnSOD possessing a Tat signal peptide and CuZnSOD is rare among cyanobacteria and in *Chroococcidiopsis* sp. CCMEE 029 it might be advantageous to cope with reactive oxygen species during desiccation, since, unlike FeSOD, they both lack the deleterious effects of the Fenton chemistry. Indeed a survey of 966 cyanobacterial SODs showed that such a coexistence occurred only in four *Chroococcidiopsis* and *Stanieria* strains and 16 filamentous cyanobacteria, belonging to *Fischerella*, *Leptolyngbya*, *Nostoc*, *Scytonema*, and *Tolypothrix* genera (not shown).

FeSOD homologs were not identified in *Chroococcidiopsis* sp. CCMEE 029, a feature that might contribute to its desiccation tolerance since iron produces highly reactive hydroxyl radical through the Fenton reaction (Aguirre and Culotta, 2012). Manganese not only lacks this deleterious effect but it might be involved in non-proteinaceous manganese-based antioxidants as reported for the desiccation- and radiation-tolerant *Deinococcus radiodurans* (Slade and Radman, 2011).

In the present work, a first insights into the involvement of the identified SOD-coding genes in the desiccation response of *Chroococcidiopsis* sp. CCMEE 029 was highlighted by their upregulation during water removal. The *sodA2.1* gene encoding the MnSOD with the Tat signal peptide for the release in the periplasm/thylakoid lumen was over-expressed already after 10 min of desiccation. The *sodA2.2* and *sodC* coding a cytoplasmic MnSOD and CuZnSOD were over-expressed after 60 min of desiccation. Furthermore *in vivo* experiments are needed to confirm the subcellular localization of the identified SODs by means of GFP tagging as well as to elucidate their role in the desiccation tolerance through gene knockout.

The capability of avoiding/limiting oxidative damage to dried subcellular components, like proteins, is crucial during the early phase of recovery of cells exposed to laboratory-air drying and space vacuum, before that DNA repair will allow *de novo* protein synthesis (Fagliarone et al., 2020; Mosca et al., 2021). Therefore, even though the contribution of non-enzymatic antioxidant systems in the desiccation tolerance of *Chroococcidiopsis* sp. CCMEE 029 remains to be deciphered, evidence were given that SODs provide a first line of defense against oxidative stress upon desiccation, thus contributing to its capability of drying without dying. The identification of the SOD-coding genes in this cyanobacterium will allow real-time monitoring of its response to oxidative stress, such as during the exposure as hydrated, metabolically active cells in future space platforms in low Erath orbit and beyond (Cottin et al., 2017).

## Data Availability Statement

The datasets presented in this study can be found in online repositories. The names of the repository/repositories and accession number(s) can be found below: https://www.ncbi.nlm.nih.gov/genbank, with accession numbers: MW422282, MW422283, MW422284, MW422285, MW422286, and MW422287.

## Author Contributions

DB and MF supervised the study and wrote the manuscript. MF, FI, AN, and GP carried out the bioinformatic analyses. CF and GP performed the gene expression analysis. All authors contributed to the article and approved the submitted version.

### Conflict of Interest

The authors declare that the research was conducted in the absence of any commercial or financial relationships that could be construed as a potential conflict of interest.

## Footnotes

^1^http://www.cbs.dtu.dk/services/SignalP/

^2^http://www.cbs.dtu.dk/services/TMHMM/
